# Great Northern Central Hospital

**Published:** 1905-03-18

**Authors:** John Dickson-Poynder, H. J. Tennant

**Affiliations:** Chairman of Committee of Management; Chairman of House Committee


Ed. The Hospital.

### GREAT NORTHERN CENTRAL HOSPITAL.

It has recently been asserted that hospitals in London minister to the needs of those capable of paying for private medical attendance. Thus, it is alleged, moneys intended for one purpose have been diverted to another, and an abuse has grown up.

A report recently presented to the management of the Great Northern Central Hospital by a sub-committee specially appointed last summer sheds some interesting light upon this statement. The sub-committee examined the *status* and means of every patient admitted to the free wards of the hospital during a period of six months—namely, from June to December 1904. The following facts are disclosed by their report:—Number of cases admitted.—Males, 281; females, 340; children under 14, 248; total, 869.

In only 15 cases, or less than two per cent., were the patients, their husbands, or parents earning 50s. a week or upwards. The highest earnings were £5 a week, in the case of a tailor's cutter suffering from cancer, who had been ill and out of work for a long time, and who died soon after admission. In four other cases the earnings exceeded £3 and were under £4 a week.

Space prevents our giving further details of these cases, but a reference to books kept at the hospital discloses in full the earnings either of the patient or the person on whom he is dependent, the age, sex, occupation, illness, and result of treatment in the hospital.

An examination of the details shows that the illness in each of the 15 cases was of a serious and urgent character, and that in none of them could the proper surgical or medical treatment have been supplied at home.

Having regard to the present system of free relief, as given by the London Hospitals, it cannot be said that this record indicates "abuse."

JOHN DICKSON-POYNDER,
Chairman of Committee of Management.
H. J. TENNANT,
Chairman of House Committee.

